# Short-term effect of electroacupuncture on rehabilitation after arthroscopic triangular fibrocartilage complex repair: a randomised study

**DOI:** 10.1186/s13018-021-02361-1

**Published:** 2021-03-24

**Authors:** Chiu-Ming Chang, Cheng-En Hsu, Yu-Chen Lee, Chen-Wei Yeh, Yung-Cheng Chiu

**Affiliations:** 1 College of Medicine, China Medical University, Taichung, 40447 Taiwan; 2 Department of Acupuncture, China Medical University Hospital, Taichung, Taiwan; 3 Sports Recreation and Health Management Continuing Studies-Bachelor’s Degree Completion Program, Tunghai University, Taichung, 407 Taiwan; 4 Department of Orthopedics, Taichung Veterans General Hospital, Taichung, Taiwan; 5 Graduate Institute of Acupuncture Science, China Medical University, Taichung, Taiwan; 6 Chinese Medicine Research Center, China Medical University, Taichung, Taiwan; 7 Department of Education, China Medical University Hospital, Taichung, 40447 Taiwan; 8 Department of Orthopedic Surgery, China Medical University Hospital, Taichung, 40447 Taiwan

**Keywords:** Triangular fibrocartilage, Acupuncture, Acupuncture therapy electroacupuncture, Acupuncture points, DASH scores, Rehabilitation

## Abstract

**Background:**

Electroacupuncture (EA) alleviates chronic pain and acute postoperative pain after several surgical procedures. However, whether EA facilitates postoperative functional recovery after arthroscopic surgery has yet to be determined. This study investigated the short-term effect of EA on a rehabilitation course after arthroscopic triangular fibrocartilage complex (TFCC) repair.

**Methods:**

Forty-two patients undergoing arthroscopic TFCC repair were randomised to an EA group (*n* = 19) or control group (*n* = 23). In the EA group, patients received EA treatment and standard active rehabilitation for 4 weeks. In the control group, patients received standard active rehabilitation for 4 weeks. At the end of the treatment and at the follow-up visit 4 weeks after the treatment, Disabilities of the Arm, Shoulder, and Hand (DASH) scores, wrist range of motion (ROM), handgrip strength, and key pinch strength were collected and analysed.

**Results:**

The EA group improved significantly than the control group in terms of DASH scores, all wrist motion arcs, and key pinch strength (*P* < 0.05) at the end of the 4-week treatment and the follow-up visit another 4 weeks later.

**Conclusion:**

Patients treated with 4 weeks of EA after the arthroscopic TFCC repair had better wrist ROM and DASH scores than patients of control group

**Supplementary Information:**

The online version contains supplementary material available at 10.1186/s13018-021-02361-1.

## Background

Arthroscopic repair for triangular fibrocartilage complex (TFCC) tear is a standard procedure with favourable outcomes because of its quicker recovery time and fewer complications than open repair [1, 2]. However, postoperative pain is a major factor hindering progress in physical therapy, consequently restricting the recovery of wrist function after surgery. Patients who do not tolerate analgesic medications may fail to derive the full benefits of stretching and strengthening exercises during critical recovery phases [3, 4]. Therefore, nonpharmaceutical treatment regimens that reduce postoperative pain can play important roles in shortening the length of a rehabilitation course and improving wrist function after surgery.

Acupuncture is a time-honoured therapy for acute and chronic pain and has considerable effects on alleviating pain after spine, shoulder, and knee surgical procedures [5–9]. Electroacupuncture (EA) is a modern variation of acupuncture that adds electricity to its needles. In clinical trials and systematic reviews, the difference in efficacy between EA and traditional acupuncture analgesia remains controversial. Nevertheless, EA offers more precise control and measurement of the electrical currents that may avoid some complications or contraindications of traditional acupuncture [10–13]. EA has recently been applied for acute pain control after open brain and knee surgery and yielded encouraging results [14–17]. However, EA has not yet been determined to help in postoperative rehabilitation courses. The purpose of our study was to investigate the effect of EA on a rehabilitation course after arthroscopic TFCC repair.

## Materials and methods

### Study design

**Fig. 1:**
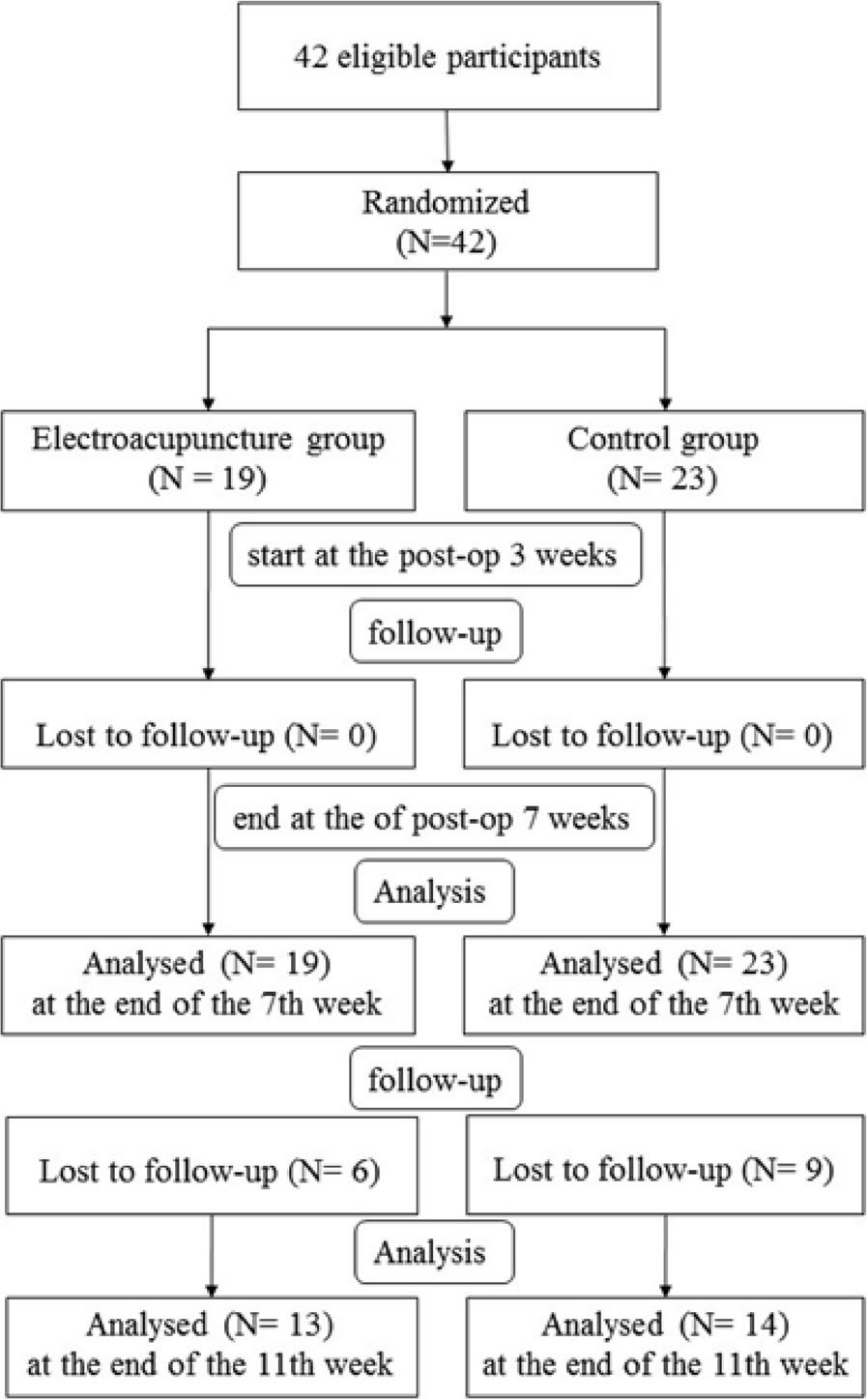
Flowchart of patient randomisation and procedures

In this single-blinded, randomised controlled study, we recruited 42 patients requiring arthroscopic repair for TFCC tears. Sealed envelopes were used to randomise the patients to either the EA or the control group. Patients in the EA group received both EA and standard active rehabilitation for 4 weeks, whereas those in the control group received only standard active rehabilitation for 4 weeks. The treatment protocol was performed for all the patients at 3 weeks after surgery. The trial was approved by the Research Ethics Committee of the China Medical University Hospital, Taichung, Taiwan (Protocol ID: CMUH108-REC2-057) and registered at ClinicalTrials.gov (identifier number: NCT04178265). Informed consent was obtained from all the patients before the trial. Figure 1 shows a flowchart of patient randomisation and procedures.

### Patients

The study included patients aged 20–60 years and received arthroscopic TFCC repair between January 2018 and January 2020. All TFCC tears were diagnosed through magnetic resonance imaging, confirmed through intraoperative arthroscopic findings and classified according to the Atzei classification. The exclusion criteria were patients with a history of wrist fracture and multiple wrist ligament injury, TFCC tears in bilateral wrists, severe cardiac arrhythmias, seizure disorders, or psychiatric diseases.

### Surgical procedure

The surgery was performed under general anaesthesia. The patient was positioned supine with shoulders abducted and the involved extremity on an arm board. A nonsterile tourniquet was employed. The hand and the forearm were draped freely after being prepared, and 10–15 lbs of traction was applied to the long and ring fingers through the finger traps and placed into the wrist traction tower. A standard 3–4 portal 1 cm distal to Lister’s tubercle was used as the viewing portal for the 2.7-mm arthroscope throughout the TFCC repair process. A 6-R portal was used as the working portal during the repair. After the completion of standard diagnostic arthroscopy, TFCC tears were identified and clearly visualised using a probe. The scar tissue was removed using a shaver to create a new bleeding surface for the formation of new scar tissue.

A 2-cm longitudinal incision was made on the ulnar side of the wrist just volar to the ECU. Blunt dissection with a right-angled clamp was used to protect the dorsal sensory branch of the ulnar nerve within the field. The dissection was carried down to the retinaculum. The instrument set for repair consisted of two 21-gauge needles for inside-out and outside-in repair with a 2-0 PDS II polydioxanone monofilament suture. With the arthroscope in the 6-R portal, the first needle loaded with the 2-0 PDS II polydioxanone monofilament suture was inserted from the 3–4 portal. The needle was then used to pierce the articular disk several millimetres radial to the edge of the tear just volar to the foveal region and out from the ulnocarpal. The 2-0 PDS was advanced out of the joint. The needle was withdrawn from the joint and pierced the articular disk again several millimetres dorsal to the first puncture site and out from the ulnocarpal joint. The 2-0 PDS was advanced out of the joint to create the first mattress suture at the foveal region using the inside–out technique. Then, the second needle loaded with a 2-0 PDS II polydioxanone monofilament suture was inserted from the lower end of the 6-R portal wound, starting approximately 1 cm proximal to the level of the ulnocarpal joint. The needle was then angled distally and radially to pierce the articular disk several millimetres radial to the edge of the tear. The 2-0 PDS was advanced into the joint, and then withdrawn with small forceps outside the joint through the 6-R portal. The third straight needle loaded with a loop of 3-0 PDS was inserted at the level of the ulnocarpal joint and then angled directly radial to come through the disk at a point dorsal to the second needle. The loop was advanced through the needle into the joint; it was then applied as a suture lasso withdrawn with small artery forceps outside the joint through the 6-R portal. The 2-0 PDS was carefully passed through the loop. The loop was removed together with the 3-0 PDS from within the joint, creating the second mattress suture. Both ends of each paired suture were sequentially rerouted such that the knot would lie directly on the retinaculum with no interposed subcutaneous tissue or potential nerve branches, after which the knots were sequentially tied. One 1.6-mm Kirschner wire was applied to maintain the reduced distal radioulnar joint in a neutral position. Standard closure of the ulnar incision and arthroscopy portals was performed. Postoperatively, the patient was placed in a short arm volar splint postoperatively for 3 weeks. The 1.6-mm Kirschner wire was removed at 3 weeks postoperatively.

### EA

**Fig. 2:**
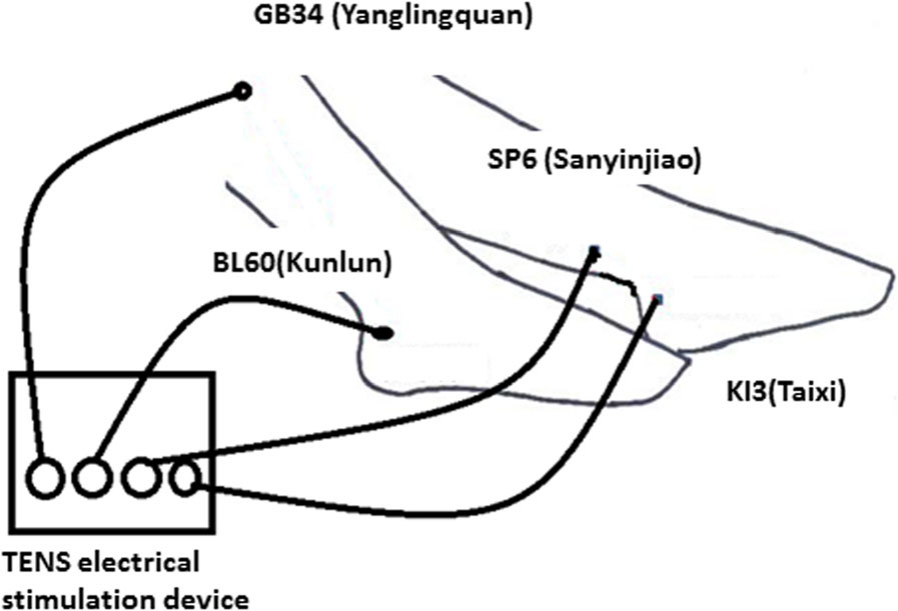
Four acupoints used, represented schematically on two legs but clinically performed only on one leg

EA treatment was applied in the 3 days after Kirschner wire removal. Four needles were inserted and the *deqi* sensation was elicited at an insertion depth of 20–30 mm, at acupoints on the contralateral lower extremity: BL60 (Bladder 60, Kunlun), GB34 (Gallbladder 34, Yanglingquan), SP6 (Spleen 6, Sanyinjiao), and KI3 (Kidney 3, Taixi; Fig. 2). The selected acupoints were cleaned with 75% alcohol before the needle insertion. The needles were single-use, disposable, sterile, stainless steel acupuncture needles, 40 mm in length and 0.22 mm in diameter (DongBang Acupuncture Inc. Boryeong, Korea). The treatment frequency was three times per week for 1 month (total = 12 sessions). Needles were connected to a constant current EA with a 2-mA intensity for 30 min using a TENS electrical stimulation device (Hometech, Tainan City, Taiwan). All acupuncture procedures were performed by a licensed Chinese medicine acupuncturist (Dr C-M Chang).

### Active rehabilitation exercises and pain management

After the 1.6-mm Kirschner wire was removed, all the patients were instructed to perform active rehabilitation exercises at home, including wrist range of motion (ROM) exercises in flexion, extension, pronation, supination, and radial and ulnar deviation three times a day for the duration of the study. All the patients were told that they could use their hands for their daily activities without restriction. All the patients received pain medication for 1 week postoperative.

### Measurements

All of the measurements, including DASH scores, wrist ROM, handgrip strength, and key pinch strength were recorded by a blinded observer before the operation, at the end of the rehabilitation exercises ± EA (7 weeks postoperatively), and again at 4 weeks after finishing the rehabilitation exercises ± EA (11 weeks postoperatively).

Handgrip strength and key pinch strength were measured with the Jamar Hydraulic Hand Evaluation Kit (Patterson Medical, Warrenville, IL, USA), involving a hydraulic hand dynamometer for the handgrip strength (HGS) and a pinch gauge for the key pinch strength (KPS). The patients were seated in the standard position with their shoulders adducted and neutrally rotated, 90° elbow flexion, forearms in a neutral position, and wrists between 0° and 30° dorsiflexion and between 0° and 15° ulnar deviation [18, 19]. The patients gripped the dynamometer and performed the test guided by standard verbal instructions. Three measurements of HGS were obtained from each patient at 30-s intervals to avoid fatigue, and the mean value of all three measurements was recorded in kilograms. A similar procedure was performed to measure KPS. For the KPS measurement, the pinch gauge was placed between the thumb and the lateral aspect of the index finger [20–22].

### Statistical analysis

Continuous variables were expressed as means ± standard deviations, and categorical variables were expressed as frequencies (%). The Shapiro–Wilk test was used to assess the normality of continuous variables. The Mann–Whitney *U* test was used to estimate the differences between the two groups. We also applied the chi-square test or Fisher’s exact test to identify differences in the binary variables. Power calculations were used to determine the sample size. For the primary outcome, we anticipated a minimal difference of interest of six points on the DASH score, with an anticipated standard deviation of six. For two groups, assuming a Type 1 error = 0.05, and power = 80%, 16 subjects per group would be needed. All statistical analyses were performed in SAS (version 9.4; SAS Institute, Cary, NC, USA), and the statistical significance level was set at α = 0.05 based on a two-sided test.

## Results

In total, 42 patients consisting of 26 (61.9%) men and 16 (38.1%) women (average age = 34.29 years; range 20–56 years) were analysed. Patients in the EA group had a significantly higher mean weight and body mass index than patients in the control group (*P* = 0.009 and 0.015, respectively). No significant differences were found in age, gender, height, Atzei classification and affected side between the two groups of patients (Table 1).

**Table 1:** Baseline characteristics of the two study groups

Characteristics^**a**^	EA group (***n*** = 19)		Control group (***n***= 23)		***P*** value^**b**^
Age, years	35.58	(12.56)	33	(10.33)	0.440
Gender					0.868
Female	7	(36.84)	9	(39.13)	
Male	12	(63.16)	14	(60.87)	
Height (cm)	168.26	(6.71)	166.39	(7.47)	0.348
Weight (kg)	72.42	(11.99)	63.30	(10.59)	**0.009**
BMI (kg/m^2^)	25.65	(4.63)	22.80	(3.06)	**0.015**
Affected side					0.635
Right	11	(57.89)	15	(65.22)	
Left	8	(42.11)	8	(34.78)	
Classification					0.868
Atzei class 2	7	(36.84)	9	(39.13)	
Atzei class 3	12	(63.16)	14	(60.87)	

^a^Categorical variables are presented as frequencies (%), and continuous variables are presented as means (SD). ^b^*P* value estimate: *P* values are calculated by the Mann–Whitney *U* test for continuous variables and a chi-square test for categorical variables

At the end of 4 weeks of active rehabilitation and EA treatment (7 weeks postoperatively), DASH scores and all wrist ROMs improved significantly in the EA group than in the control group (*P* < 0.05). Patients in the EA group also had higher KPS than those in the control group (*P* < 0.05). No significant difference was found in HGS between the two groups (Table 2).

**Table 2:** Hand function of the two groups at the end of the treatment (7 weeks postoperatively)

Measurements	EA group (***n*** = 19)		Control group (***n*** = 23)		***P*** value^**b**^
DASH (points)	18.92	(11.68)	45.45	(9.81)	< 0.001
Wrist ROM, degree					
Flexion/extension arc	100.84	(17.56)	75.74	(22.27)	**0.001**
Pronation/supination arc	163	(18.13)	117	(12.36)	**< 0.001**
Radial/ulnar arc	51.58	(8.9)	30.74	(9.38)	**< 0.001**
Handgrip strength, %	87	(79)	67	(37)	0.211
Key pinch strength, %	93	(32)	87	(32)	**0.018**

Categorical variables are presented as frequencies (%) and continuous variables are presented as means (SDs). Data are presented as means ± standard deviations. ^b^*P* value estimate: *P* values are calculated by the Mann–Whitney U test for continuous variables and a chi-square test for categorical variables

At the follow-up visit 4 weeks after the end of active rehabilitation and the EA treatment (11 weeks postoperatively), DASH scores and radial and ulnar motion arc remained significantly improved in the EA group than in the control group (*P* < 0.05). However, no significant difference was noted in flexion and extension motion arcs, pronation and supination motion arcs, HGS, or KPS (Table 3). No infection or other complication was observed during the study period.

**Table 3:** Hand function at the follow-up visit 4 weeks after the treatment (11 weeks postoperatively)

Measurements	EA group (***n*** = 13)		Control group (***n*** = 14)		***P*** value^**b**^
DASH (points)	12.58	(8.89)	24.91	(9.45)	0.003
Wrist ROM, degree					
Flexion/extension arc	112.09	(15.23)	107.75	(24.67)	0.600
Pronation/supination arc	170	(15.95)	148.83	(32.01)	0.076
Radial/ulnar arc	56.73	(6.78)	41.5	(12.15)	**0.003**
Handgrip strength, % (compared with normal side)	82	(28)	74	(26)	0.951
Key pinch strength, % (compared with normal side)	91	(23)	94	(23)	0.757

Categorical variables are presented as frequencies (%), and continuous variables are presented as means (SDs). Data are presented as means ± standard deviations. ^b^*P* value estimate: *P* values are calculated by the Mann–Whitney *U* test for continuous variables and a chi-square test for categorical variables

**Fig. 3:**
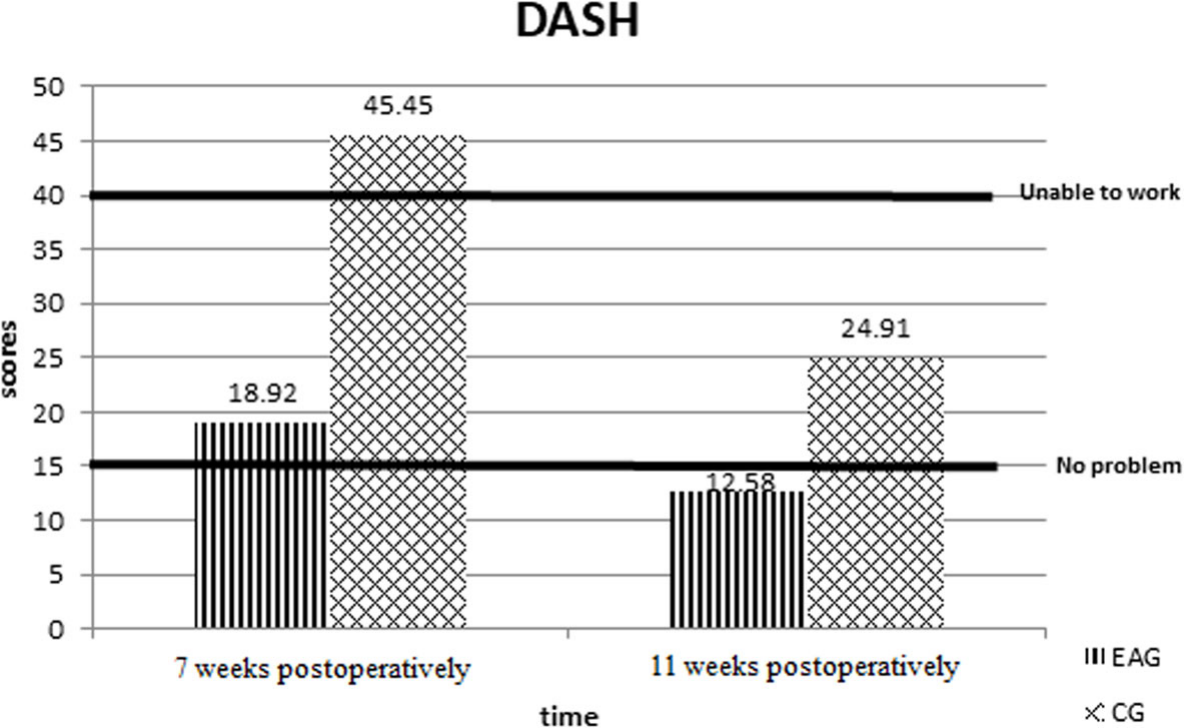
Cutoff points to judge clinically meaningful improvements. At the end of 4 weeks of therapy, the DASH score levels were between the levels of ‘no problem’ and ‘unable to work’ in the EA group, but they increased beyond the levels of ‘unable to work’ in the control group. At the follow-up visit 4 weeks after therapy, the DASH score levels were already lower than the level of ‘no problem’ in the EA group, but they remained high in the control group

In the EA group, the DASH score reached 18.92 at the end of the 4-week treatment (7 weeks postoperatively), which was significantly lower than that of the control group (DASH score = 45.5) and was also much lower than the cutoff point for ‘unable to work’ according to the interpretation of DASH scores [28]. At the follow-up visit 4 weeks after the treatment (11 weeks postoperatively), DASH scores in the EA group continued to be significantly lower than in the control group but also reached the cutoff point for ‘no problem’ according to the interpretation of the DASH scores (Fig. 3).

## Discussion

In our study, we determined whether EA accelerates rehabilitation after arthroscopic TFCC repair. Our main findings were that patients treated with EA had significantly improved DASH scores and wrist ROMs in the early rehabilitation period than those who had only received active rehabilitation after arthroscopic TFCC repair.

The safety and effectiveness of EA in reducing acute postoperative pain following several surgical procedures including open heart surgery, pneumonectomy, hip replacement, hysteroscopic surgery, and nasal endoscopic sinus surgery have been documented [23–27]. Our study indicated that in combination with active rehabilitation exercise, EA is a safe and effective procedure for wrist function and ROM recovery in postoperative rehabilitation courses following arthroscopic TFCC repair. The DASH score of EA group reached 18.92 at the end of the 4-week treatment (7 weeks postoperatively), which was much lower than the cutoff point for ‘unable to work’ according to the interpretation of DASH scores [28] (Fig. 3). These results suggest that the EA had a short-term positive effect on DASH score after arthroscopic triangular fibrocartilage complex repair. However, the long-term effects still need to be elucidated. Our study recorded detailed DASH scores and wrist ROMs in the early stages after the arthroscopic TFCC repair, which previous studies have rarely reported. In the literature, functional scores and wrist ROMs after arthroscopic TFCC repair are typically reported 24–36 months postoperatively. Studies have reported DASH scores of 10.2–15.0, grip strengths of 89–103%, and flexion and extension arcs of 92–98% in the non-operative hand [29–31]. In our study, the EA group exhibited a DASH score comparable to the DASH scores in previous studies at very early stage after the operation (DASH score was 12.58 at 11 weeks postoperatively). Our study demonstrated that the EA had a short-term positive effect on the rehabilitation course in the very early stage after arthroscopic triangular fibrocartilage complex repair. Although the central mechanisms differed between acupoints and non-acupoints, the penetration of a needle through the skin could produce a physiological effect [32]. Furthermore, even with the same stimulus mode, different acupoints have different responses in the brain and different therapeutic effects [33, 34]. In our study, the four acupoints used for EA were all on the contralateral lower extremity rather than the upper extremity. The reason for selecting these acupoints was to avoid the concern of wound infection, reduce pain, and improve wrist ROM during the EA treatment [4]. We believed that the main effect of EA was to decrease the pain intensity after the operation, thus encourage patients to cooperate with rehabilitation plan and had a more favourable DASH score.

There were several limitations to our study. First, we did not include a sham group in our study’s design. However, the use of a sham group may not be superior to that of control groups [35]. Second, we did not record pain scores during the activities. However, a pain evaluation was included in the second part of the DASH score. The significant improvement in DASH scores also indicates a considerable decrease in pain. Third, the case number of our study is relatively small, and the follow-up time of our study is very short. The results of our study should be confirmed by future studies with a larger population and a longer follow-up duration.

## Conclusion

Patients treated with 4 weeks of EA after the arthroscopic TFCC repair had better wrist ROM and DASH scores than patients of control group

## Supplementary Information

**Additional file 1.** CONSORT Checklist.

## Data Availability

The datasets used and/or analysed during the current study are available from the corresponding author on reasonable request.

## Abbreviations

EA: Electroacupuncture
TFCC: Triangular fibrocartilage complex
DASH: Disabilities of the Arm, Shoulder, and Hand
ROM: Wrist range of motion
HGS: Handgrip strength
KPS: Key pinch strength
TENS: Transcutaneous electrical nerve stimulator
